# 1864. Recruitment of Participants for Qualitative Research in Marginalized Populations

**DOI:** 10.1093/ofid/ofad500.1692

**Published:** 2023-11-27

**Authors:** Yamini Adusumelli, Mary Tabatneck, Susan N Sherman, Gabriella S Lamb, Vishakha Sabharwal, Don Goldmann, Alexandra Epee-Bounya, Jessica Haberer, Thomas J Sandora, Jeffrey I Campbell

**Affiliations:** Boston University School of Medicine, Boston, Massachusetts; Boston Children’s Hospital, Boston, Massachusetts; SNS Research, Cincinnati, Ohio; Boston Children's Hospital, Boston, Massachusetts; Boston University, Boston, Massachusetts; Harvard Medical School, lexington, Massachusetts; Boston childrens' Hospital, Boston, Massachusetts; Harvard Medical School, lexington, Massachusetts; Boston Children's Hospital, Boston, Massachusetts; Boston Medical Center, Boston, Massachusetts

## Abstract

**Background:**

There is limited understanding of barriers to participation in pediatric qualitative research, particularly for studies seeking to understand diseases primarily affecting marginalized and hard-to-reach populations.

**Methods:**

We conducted a retrospective cohort study of participants and non-participants invited to enroll in a qualitative study of pediatric tuberculosis (TB) infection care. Potential participants in the initial study were adult caregivers of children diagnosed with TB infection between 2016-2021 in Boston. Most children were immigrants or children of immigrants, and all caregivers were English- or Spanish-speaking. We sent letters in English and Spanish to patients’ primary caregivers, and followed up with up to 2 phone calls. Enrolled participants completed a virtual qualitative interview. We compared characteristics of individuals who participated vs did not participate, and summarized reasons for non-participation.

**Results:**

Of 61 invited caregivers, 19 (31%) enrolled in the initial qualitative study. Table 1 compares characteristics of participants and non-participants. The primary reason for non-participation was inability to contact caregivers (n=27, 64%). A total of 11 (26%) caregivers declined participation, and 4 (10%) participants were unable to join or complete interviews after initially agreeing to participate. Non-completion of TB infection care was significantly associated with non-participation in the qualitative study (P=0.049). We did not observe significant differences by language, insurance, relationship of caregiver to patient, or time from diagnosis to study invitation between participants and non-participants.

**Figure:**
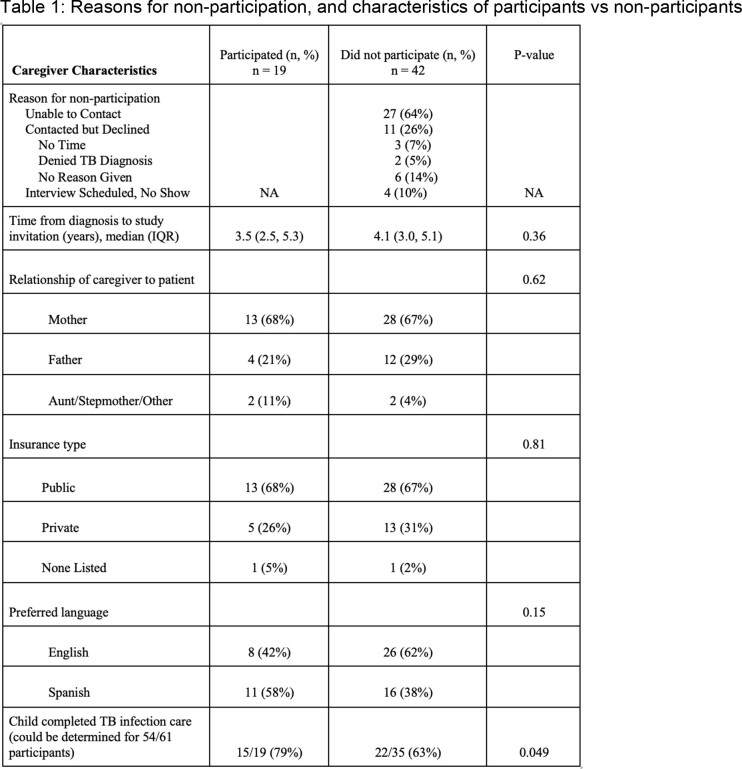

**Conclusion:**

Inability to contact potential participants, participants’ lack of time, and lack of shared illness understanding posed barriers to recruitment in this qualitative study. Our findings suggest that alternative contact strategies (e.g. email, text messages), use of short interviews, and strategies to rapidly communicate illness scripts should be studied to improve inclusion in similar studies. While we were able to enroll participants who had not completed TB infection care, additional methods are needed to obtain qualitative perspectives from individuals who disengage from care.

**Disclosures:**

**Jessica Haberer, MD, MS**, Merck: Advisor/Consultant

